# Virtual Ice Cream Rounds: Addressing medical clerk wellness during COVID-19

**DOI:** 10.36834/cmej.70251

**Published:** 2020-09-23

**Authors:** Hannah Kearney, Jorin Lukings

**Affiliations:** 1 Michael G. DeGroote School of Medicine, Niagara Regional Campus, Ontario, Canada

## Implication Statement

Ice Cream Rounds (ICRs) are used by residency and clerkship programs across Canada to provide trainees with a safe space to debrief difficult situations, connect with peers, and promote wellness. At the Niagara Regional Campus of the Michael G. DeGroote School of Medicine, ICRs are held often and are well-received by learners. In light of the COVID-19 pandemic, we adapted and facilitated the first virtual ICR. Post-session, students reported an increase in nearly all domains of their perceived wellness. Due to the uncertainty associated with this unprecedented time, this event could help learners at other institutions support each other.

## Déclaration des Répercussions

Les rondes de crème glacée (RCG) sont utilisées par les programmes de résidences et de stages médicales partout au Canada pour offrir aux stagiaires un espace sûr pour faire le point et réfléchir sur des situations difficiles, entrer en contact avec des collègues et promouvoir le bien-être. Au campus régional de Niagara de l’école de médecine Micheal G. DeGroote, on tient régulièrement des RCGs et elles sont bien reçues par les apprenants. Dans le contexte de la pandémie de COVID-19, nous avons adapté et animé la première RCG virtuelle. Après la séance, les étudiants ont rapporté une augmentation de leur bien-être perçu dans presque tous les domaines. En raison de l’incertitude associée à cette période sans précédent, cet évènement pourrait aider les apprenants d’autres institutions à s’entraider.

## Introduction

Clerkship is a stressful time for medical learners. This transitional point in medical education is associated with longer work hours, new responsibilities, novel clinical situations, and the stress of residency applications.^1^ Students are often separated from their classmates and have fewer formal opportunities for support. Ice Cream Rounds (ICRs)—an initiative adopted by medical programs across Canada—provide an opportunity for trainees to discuss challenging clinical encounters, share coping strategies, and promote self-care.^2^

The Niagara Regional Campus (NRC) of the Michael G. DeGroote School of Medicine facilitates bi-monthly ICRs for medical clerks. These confidential sessions are run with student affairs support and provide a space for students to discuss challenges and successes while reconnecting with peers and enjoying ice cream. Concerned that the COVID-19 pandemic may leave insufficient opportunities for peer support and wellness promotion during a stressful time, we created Virtual Ice Cream Rounds (VICRs).

## Methods

Medical clerks in the class of 2021 initially received a needs assessment. Twenty of 28 invited students completed the survey. Eighty percent of respondents believed ICRs would be beneficial for their wellness and were interested in participating. When asked about COVID-19, 60% of respondents indicated increased anxiety and reported that the pandemic had negatively influenced aspects of their physical, mental, social, intellectual, or occupational wellness.

All 28 students received an invitation to an optional 1-hour VICR on Zoom one week later. The cohort wellness representative (HK) and Student Affairs Director (JL) co-facilitated the session. The VICR began with a review of safe space principles and then became a semi-structured discussion.^3^ Afterward, attendees completed a follow-up survey. This project was exempted from ethics review by HiREB.

## Results

**Figure 1:**
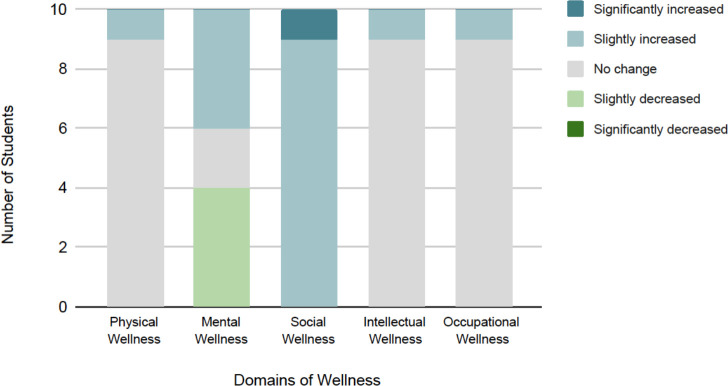
Impact of VICRs on students’ perceptions of personal wellness domains. *Physical wellness was described as managing health conditions, sleeping, eating, and physical activity. Mental wellness was defined as mood, anxiety, and managing mental health conditions. Social wellness encompassed feelings of inclusion and connectedness to others. Intellectual wellness was composed of ability to continue towards achieving academic goals and learning. Occupational learning was defined as safety in the learning environment*.

Sixteen of 28 invited students attended the VICR. Ten attendees completed the post-session survey. All respondents felt the VICR was a safe space to share concerns, and 90% reported increased connection to their peers. Benefits from the VICR included: collegiality and support amongst peers (80%), hearing about coping strategies (80%), and having a safe space to voice frustrations (80%). All responders also reported they would recommend ICRs to their peers. Unexpectedly, 40% of attendees stated that participating in the VICR decreased their perceived mental wellness, despite reporting positive changes in all other wellness domains (Figure 1).

## Discussion

This initiative demonstrates that despite being online (and sans ice cream), VICRs can serve as a safe space for clerks to share difficulties and support each other. Our finding that 80% of students felt a sense of collegiality and support among their peers affirms prior work suggesting that ICRs are a helpful forum for peer support, while also demonstrating the efficacy of an online format.^2^

Although unexpected, we infer that the decrease in mental wellness reported by some attendees may be due to the reflective process enabling students to identify personal struggles they had previously been ignoring.^4^ To better understand this finding, future ICRs should investigate the impact of group reflective practice on students’ perceived wellness.

Limitations of this study include small sample size, convenience sampling, and inability to capture the perspectives of students who did not partake.
